# COVID-19 Modeling in Saudi Arabia Using the Modified Susceptible-Exposed-Infectious-Recovered (SEIR) Model

**DOI:** 10.7759/cureus.10452

**Published:** 2020-09-14

**Authors:** Naim Ahmad

**Affiliations:** 1 Information Systems, King Khalid University, Abha, SAU

**Keywords:** coronavirus disease (covid-19), compartment epidemiology model, susceptible-exposed-infectious-recovered (seir) model, modified seir, social distancing, testing, infectiousness of exposed and infectious compartments, threshold herd immunity, basic reproduction number, saudi arabia

## Abstract

The coronavirus disease 2019 (COVID-19) pandemic has created unprecedented healthcare emergencies across the globe. The World Health Organization (WHO) has proposed social distancing (SD) as a prudent measure to contain the pandemic and, hence, governments have been enacting lockdowns of varied nature. These lockdowns, causing economic and social strain, warrant the development of quantitative models to optimally manage the pandemic. Similarly, extensive testing aids in early detection and isolation, hence containing the spread of the pandemic. Compartment epidemiology models have been used extensively in modeling such infectious diseases. This paper attempts to utilize the modified Susceptible-Exposed-Infectious-Recovered (SEIR) model incorporating the SD, testing, and infectiousness of exposed and infectious compartments to study the COVID-19 pandemic in Saudi Arabia. Saudi Arabia has put restrictions on the movement of people in different phases to ascertain SD. Time-dependent parameters based on the timeline of restrictions and testing in Saudi Arabia have been introduced to capture SD and testing. The arrived model has been validated through statistical tests. The $R_{0}$ (R naught), basic reproduction number, value has ranged between 0.6014 and 2.7860 with an average of 1.4904 and currently holds at 0.8952. In the absence of SD and testing measures, the model predicts the threshold herd immunity to be 69.31% and $R_{0}$ value as 3.26. Further, scenario analysis has been conducted for alleviating the SD measure. The results show that early lifting of all restrictions may undo all efforts in the containment of the COVID-19 pandemic. The outcome of results will help policymakers and medical practitioners prepare better to manage the pandemic and lockdown.

## Introduction

The current coronavirus disease 2019 (COVID-19) pandemic has spread from the Chinese city of Wuhan. It mainly causes the common cold and could cause severe acute respiratory syndrome and may develop into fatal pneumonia [1]. It transmits from person to person through direct and indirect contact, the fecal-oral route, and respiratory aerosolized droplets [2-3]. It was first reported in Wuhan in late December 2019, and the first case in Saudi Arabia was reported on March 2, 2020 [1]. As of August 19, 2020, in Saudi Arabia, there are a total of 303,973 confirmed cases, out of which 275,476 people have recovered and 3,548 died, leaving 24,949 active cases [4].

The COVID-19 pandemic has created restrictions on the movement of people and everyday activities, which is termed as social distancing (SD). As the World Health Organization (WHO) endorses physical distancing, wearing masks, washing hands with soap, and cleaning hands with alcohol-based hand rubs as some of the solutions to contain the pandemic until an effective drug or vaccine is available. This has resulted in lockdowns and severe strains on the economic activities and the mental and psychological well-being of many. Therefore, to strike a balance between healthcare and economy, decision-makers need a quantitative model to predict the pandemic in order to ease lockdowns, resume economic activities, and protect the mental and psychological well-being of people. Compartment epidemiology models have been widely used to quantify the trends of such infectious diseases. This study aims at using such a compartment epidemiology model to analyze the dynamics of the COVID-19 epidemic in Saudi Arabia.

The base compartment epidemiology model is Susceptible-Infectious-Recovered (SIR) [5] and the extension of it is Susceptible-Exposed-Infectious-Recovered (SEIR). To study the dynamics of the COVID-19 epidemic, several modified SEIR models have been used to incorporate various measures, especially social distancing, testing, public responses, and mobility restrictions [2,6-9]. The modified models have a large number of parameters that increase the complexity. Incorporation of social distancing is essential and Hubbs [10] has used a single parameter to incorporate SD. Further, as the measure of social distancing varies with time, hence, De Falco et al. have introduced dynamic SD [11]. Dur-e-Ahmad et al. [9] incorporate the infectiousness among the exposed compartment similar to that of the infectious compartment as identified by He et al. [12]. And Berger et al. have modified the model with a testing parameter [7]. The objective of this paper is to model the COVID-19 pandemic in Saudi Arabia using SEIR, including SD, testing, and the infectiousness of exposed and infectious populations. For the simulation of the mathematical model, the Python language has been used and results are validated with statistical analysis. Further, the scenario analysis has been done to calculate threshold herd immunity and to study the effects of the alleviation of restrictions.

## Materials and methods

Base SEIR model

The compartment epidemiology model SEIR divides the population of study into four compartments: susceptible $S(t)$, exposed $E(t)$, infectious $I(t)$, and recovered $R(t)$. The susceptible compartment represents a population that is prone to COVID-19, whereas the exposed population has acquired coronavirus but is asymptomatic. An infectious population has symptoms of coronavirus such as fever, cough, and fatigue and may infect the susceptible population. Finally, the recovered compartment represents Individuals that have either recovered or died. Mathematically different compartments are governed by following differential equations (1-4). It is assumed that the total population ($N = S(t)+E(t)+I(t)+R(t)$) remains fixed and recovered individuals remain immune to the disease during the study period.

$$\frac{dS}{dt}= -\beta\cdot I\cdot \frac{S}{N}\qquad(1)$$

$$\frac{dE}{dt}= \beta\cdot I\cdot \frac{S}{N}-\alpha\cdot E\qquad(2)$$

$$\frac{dI}{dt}= \alpha\cdot E-\gamma\cdot I\qquad(3)$$

$$\frac{dR}{dt}= \gamma\cdot I\qquad(4)$$

The parameter $\beta$ represents the average contact rate and $\alpha$ and $\gamma$ represent the inverse of the central measures of incubation period and infectious period. There is also one important index R Naught, $R_{0} =\frac{\beta}{\gamma}$, known as the basic reproduction number that signifies the number of susceptible individuals that will get infected by an infectious individual. The value of $R_{0}$ needs to be less than one for a pandemic to die out.

Modified SEIR model

In the SEIR with SD model, one more parameter $\rho$ is introduced to represent SD that varies between 0 (ideal isolation) and 1 (no SD) [10]. And in a dynamic SD case, the value of $\rho$ will vary with time based on the SD measure variations [11]. Similarly, Dur-e-Ahmad et al. have incorporated $\eta_{1}$ and $\eta_{2}$ to represent the relative infectiousness of exposed and infectious compartments, respectively [9]. Further, there are studies that have added parameter $\tau$ to represent testing [7]. Hence, a composite parameter to represent net measures is computed as $\nu = \rho - \tau$, and mathematically, the positive part of this parameter ($\nu^{+}$) is used, meaning for negative values, it will be zero. Therefore, the mathematical formulation of the modified SEIR model adopted for the current study changes equations (1,2) as follows, whereas the equations (3,4) remain the same.

$$\frac{dS}{dt}= -\beta\cdot \nu^{+}\cdot\left ( \eta_{1}\cdot E +\eta_{2}\cdot I\right ) \cdot \frac{S}{N}\qquad(5)$$

$$\frac{dE}{dt}= \beta\cdot \nu^{+}\cdot\left ( \eta_{1}\cdot E +\eta_{2}\cdot I\right ) \cdot \frac{S}{N}-\alpha\cdot E\qquad(6)$$

Basic reproduction number ($R_{0}$) for the modified SEIR model

The basic reproduction number ($R_{0}$) is “defined as the expected number of secondary cases produced by a single (typical) infection in a completely susceptible population” [13]. A next-generation matrix may be used to drive the equation of $R_{0}$​​​ [14]. Using equations (6,3), new functions $F$ and $V$ may be defined to represent the rate of new infections and the rate of transfer in and out of exposed and infectious compartments. Therefore, the matrices $F$ and $V$ are defined, as shown in equations (7,8).

$$F=\begin{bmatrix} \beta\cdot \nu^{+}\cdot \eta_{1} &\beta\cdot \nu^{+}\cdot \eta_{2} \\ 0 & 0 \end{bmatrix} \qquad(7)$$

$$V=\begin{bmatrix} \alpha &0 \\ -\alpha & \gamma \end{bmatrix} \qquad(8)$$

And the next generation matrix is defined as $G = F\cdot V^{-1}$. The largest eigenvalue of $G$ (the spectral radius) gives $R_{0}$, as given in equation (9).

$$R_{0}= \beta\cdot \nu^{+}\cdot\left ( \frac{\eta_{1}}{\alpha} +\frac{\eta_{2}}{\gamma}\right)\qquad(9)$$

Model parameters and values

There are a total of seven parameters $\alpha$, $\beta$, $\gamma$, $\rho$, $\tau$, $\eta_{1}$, and $\eta_{2}$, and one derived parameter $\nu^{+}$. $\alpha$ and $\gamma$ are disease-specific and calculated with incubation and infectious periods. $\beta$ signifies the contact rate and is modified by $\rho$ and $\tau$ accounting for social distancing and testing, respectively, or by net measures $\nu^{+}$. $\eta_{1}$ and $\eta_{2}$ are modification parameters for the relative infectiousness of exposed and infectious populations.

The incubation period ($\frac{1}{\alpha}$): The time period during which the exposed people become infectious and symptomatic is called the incubation period. The incubation period ranges between two and 14 days [15]. Generally, the mid-value of 7 days of the incubation period is considered but in the case of Saudi Arabia, the median incubation period is identified as six days [16]. The inverse of the incubation period is known as the incubation rate.

Infectious period ($\frac{1}{\gamma}$): The time period during which an individual remains infectious and thereafter recovers or dies. This period is given between zero to 10 days [17], three days or seven days [18], and 2.9 days [19]. The inverse of the infectious period is known as the recovery rate. The value of this parameter has been taken within the acceptable range and in conjunction with model fitting in simulation.

**Figure 1:**
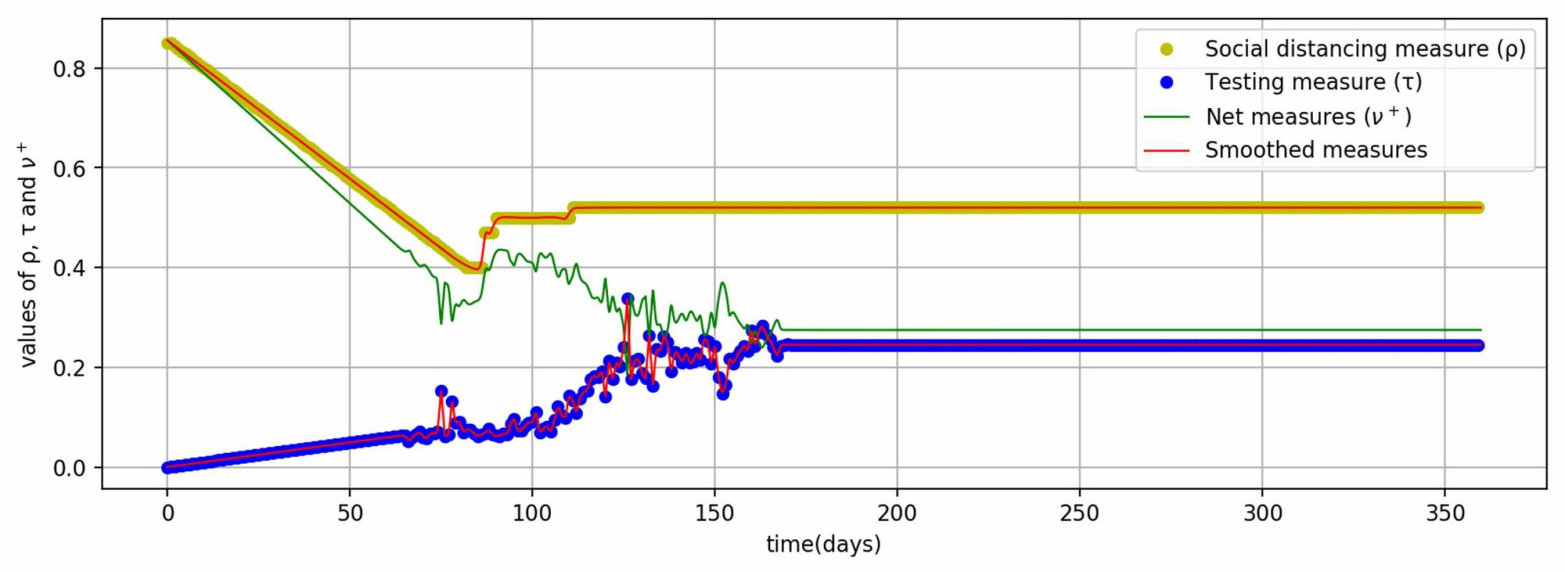
Values of parameters of social distancing, testing, and net measures

Net measures ($\nu^{+}$): The net measures ( $\nu^{+}$ ) is mathematically the positive part of the difference of social distancing ($\rho$) and testing ($\tau$). The social distancing ($\rho$) measures are implemented differently by countries and vary with time. Saudi Arabia firstly suspended overseas Umrah visitors on February 26, 2020 [20]. The first case of COVID-19 was detected on March 2, 2020 [16]. Thereafter, restrictions of a different nature were implemented such as curfews, classroom learning at schools and institutions, closure of private and public sectors, inter-city movements, hot-spot isolation, suspension of sports events, restrictions on public gatherings, prayers in Masjids, suspension of domestic air and road transport, and others. The intensity of these measures was at the peak during March 23-27, 2020, for the Eid festival by a 24 hours nationwide curfew. After this relaxation period started in three phases, May 28-30, 2020, May 31 - June 20, 2020, and June 21, 2020, onward. During the first phase, curfew was relaxed between 6 AM and 3 PM, in the second phase, it was extended to 8 PM, and in the third phase, it was relaxed completely. And all of the economic activities have been allowed with precautionary measures, such as face masks, sanitization, and physical distancing. But, still, international flights, overseas Umrah, and school and colleges are closed and precautionary measures are being observed. Based on these measures, a time-varying SD parameter has been defined, as shown in Figure 1. The testing data for Saudi Arabia has been taken from the Saudi Ministry of Health [4] and the Oxford COVID-19 Government Response Tracker [21] and indexed between 0 and 1 to compute $\tau$ (Figure 1). Both $\rho$ and $\tau$ values have been chosen to represent the SD restrictions and testing data in conjunction with model fitting in simulation. Figure 1 also shows the values of net measures ($\nu^{+}$).

Contact ratio ($\beta$), infectiousness of exposed and infectious populations ($\eta_{1}$ and $\eta_{2}$): The values of $\eta_{1}$ and $\eta_{2}$ are taken in the range of 0.4 to 1 and may be estimated through optimization techniques [9]. The value of $\eta_{2}$ is higher than the value of $\eta_{1}$. The value of contact ratio ($\beta$) is also estimated through optimizations techniques [8]. The value of $\eta_{2}$ has been taken near 1 and the values of $\eta_{1}$ and $\beta$ have been derived through simulation and optimization techniques.

## Results

Simulation

The model represented by ordinary differential equations (5,6,3,4) is utilized for the simulation of the pandemic in Saudi Arabia. Coding was done in the Python language [22] in the environment of Jupyter Notebook [23]. The equations were solved with the help of the function solve_ivp from the scipy.integrate sub-package. Further, the minimize function from the scipy.optimize sub-package was used to minimize the difference between predicted and reported values of total cases to estimate $\beta$ and $\eta_{1}$. The values of other parameters were given as defined previously such as $\alpha=1/6, \ \gamma=1/4.9\, and \ \eta_{2}=.9$. For the values of $\rho$ and $\tau$, discrete events of SD and testing data were smoothed out slightly using the UnivariateSpline function with a smoothing factor of .001 (Figure 1). Thereafter, the values of $\nu^{+}$ were computed from the smoothed values of $\rho$ and $\tau$. The initial values for solving the ordinary differential equations were as follows $I(0) = 1$ (cases on first day), $E(0) = 5$ (assumed that five times individuals are exposed on first day), $R(0)= 0$ (recovered on first day) and $S(0)=34,813,861$, as the total population of Saudi Arabia is 34,813,867. The Levene statistical tests were performed to validate model results against the epidemic reported data from the Saudi Ministry of Health [4].

Model results

**Figure 2:**
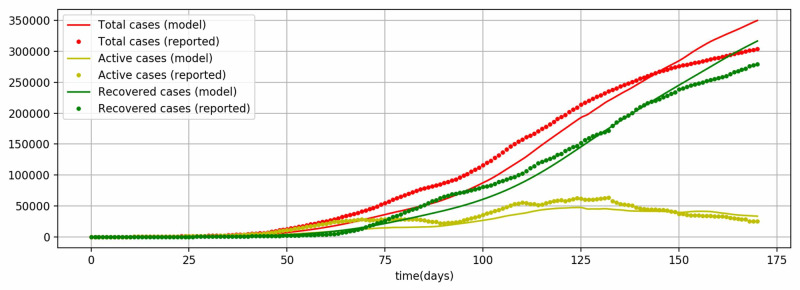
Model fitting for total cases, recovered cases, and active cases up to August 19, 2020

The resultant model identified the values of $\beta$ and $\eta_{1}$ to be 0.4016 and 0.6170 by fitting the values of cumulative total cases against the reported cumulative total cases up to August 19, 2020 (Figure 2). The Levene test (statistic=0.0229, p-value=0.8798) shows that there is no significant difference between model values and reported values. Further, in Figure 2, it is evident that the model is fitting closely with the cumulative recovered cases as well. As the Levene test (statistic=0.0308, p-value=0.8608) confirms that there is no significant difference between model values and reported values. Similarly, the cumulative active cases that include exposed and infectious populations fit closely and the Levene test (statistic=0.5088, p-value=0.4758) proves the same. The results of the simulation show a very significant model fitting, as all the three publicly reported data, such as total cases, recovered cases, and active cases, show no significant difference from the respective model values.

## Discussion

Current scenario

**Figure 3:**
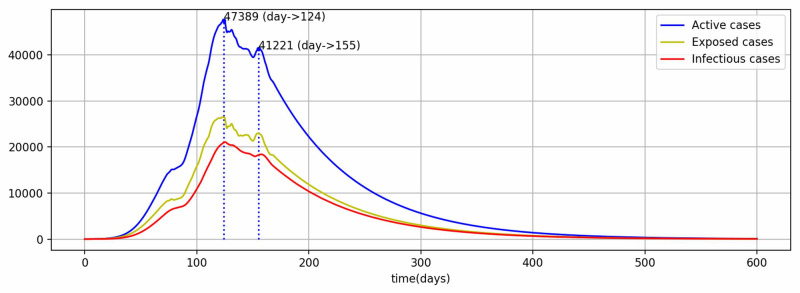
Model projected results in the current scenario for active, exposed, and infectious cases

The model results in the current scenario of restrictions and testings depict that there are multiple peaks for active cases (Figure 3). It identifies two visible peaks in the period of 20 days of neighborhood data settings to identify the local maxima. This pattern also closely resembles the reported data although the absolute values differ with the reported data within statistical non-significant limits. The $R_{0}$ value has ranged between 0.6014 and 2.7860, with an average of 1.4904, and currently holds at 0.8952. As the current value of $R_{0}$ is less than one, the pandemic is expected to decline and the peak has already passed (Figure 3).

Herd immunity

**Figure 4:**
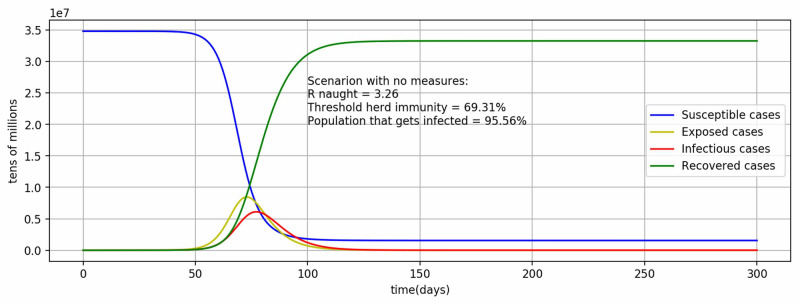
Model results in the scenario of no measures to compute threshold herd immunity

It is important to achieve herd immunity for the pandemic to decline in the absence of any measures. As of now, there is no proven vaccine for COVID-19, therefore, herd immunity can only be achieved when a substantial proportion of the population has recovered with the assumption that the recovered become immune to COVID-19. Therefore, this scenario removes the SD and testing measures and recomputes the model results with the parameter values identified in the fitted model previously. The $R_{0}$ value has come out to be 3.26 and threshold herd immunity ($1-1/R_{0}$) will be achieved at 69.31% of the population (Figure 4). Further, the pandemic will infect 95.56% of the population by the end.

Scenarios with varied SD measures

**Figure 5:**
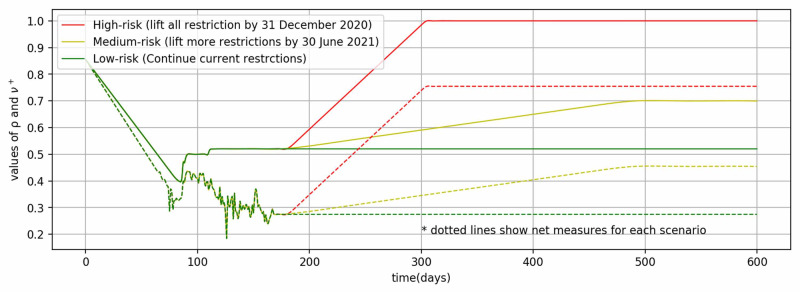
Social distancing and net measures for different scenarios

Further, the SD measures were varied for two possibilities and compared with the current measures (low-risk scenario). The first possibility is gradual full normalization by December 31, 2020, and termed as a high-risk scenario. And the second possibility could be gradual partial normalization by June 30, 2021, the expected date of vaccine (medium-risk scenario), whereas COVID-19 testing is assumed to continue at the same level. The values of $\rho$ and $\nu^{+}$ for the above-mentioned three scenarios are shown in Figure 5.

**Figure 6:**
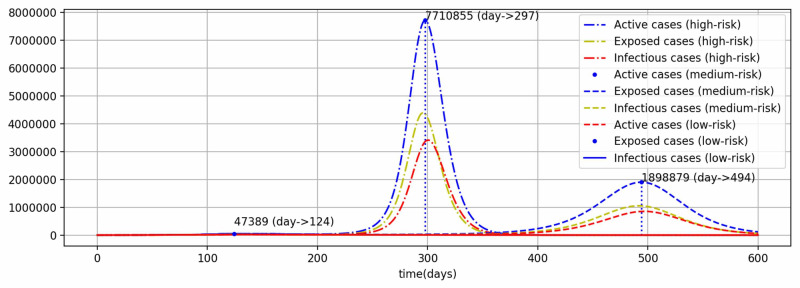
Model results in varied measures of social distancing scenarios

The peak total active cases are 47,389, 1,898,879 and 7,710,855 for low-risk, medium-risk, and high-risk scenarios, respectively (Figure 6). These peak values are supposed to be achieved on the 124^th^ day (July 4, 2020), 494^th^ day (July 9, 2021), and 297^th^ day (December 24, 2020) for the respective scenarios. In the low-risk scenario, the peak has already passed at a relatively very low value of 47,389 for active cases, the current scenario. In the second scenario of medium-risk, the peak is flattened and delayed beyond the arrival of the expected vaccine date, although the peak value is almost 1.9 million of active cases, which is a huge number to be managed. The third scenario of high risk will bring a very high peak value of almost 7.7 million active cases before the end of this year and way before the expected vaccine date.

## Conclusions

This study has modeled the COVID-19 pandemic using a modified version of the base compartment epidemiology model of SEIR. The modification was achieved by the introduction of four parameters accounting for the social distancing, testing, and infectiousness of exposed and infectious populations. The mathematical equations corresponding to the modified model were simulated in a Python environment. The optimization technique was used to fit the model with reported cases by the varying contact rate and infectiousness of the exposed population. The remaining model parameters were chosen within the acceptable range and in conjunction with model fitting. The statistical analysis shows a good model fit, as the model results for total cases, recovered cases, and active cases have no significant difference with those of reported cases. As per model results, the peak has already passed and the pandemic is expected to decline with current measures in place. Hence, the current measures have been proven to be very effective in Saudi Arabia. The model has been run on different scenarios for social distancing to predict the possible outcomes. The results indicate that early complete normalization efforts may undo the successful containment of the pandemic. Therefore, the decision to alleviate restrictions has to be well worked with other mechanisms such as increasing the testing capacity and throughput and the availability of effective drugs and vaccines. Nonetheless, due to unavoidable assumptions and the exponential nature of functions for all categories of cases, actual numbers may vary from the model results and care should be taken in interpreting the results.

## References

[1] Algaissi AA, Alharbi NK, Hassanain M, Hashem AM (2020). Preparedness and response to COVID-19 in Saudi Arabia: building on MERS experience. J Infect Public Health.

[2] Aletreby W, Alharthy A, Faqihi F (2020). Dynamics of SARS-CoV-2 outbreak in the Kingdom of Saudi Arabia: a predictive model. Saudi Crit Care J.

[3] Nouri-Vaskeh M, Alizadeh L (2020). Fecal transmission in COVID-19: a potential shedding route. J Med Virol.

[4] (2020). Ministry of Health. Protection from Covid-19. https://covid19.moh.gov.sa/.

[5] Kermack WO, McKendrick AG (1927). A contribution to the mathematical theory of epidemics. Proc R Soc A.

[6] Fang Y, Nie Y, Penny M (2020). Transmission dynamics of the COVID‐19 outbreak and effectiveness of government interventions: a data‐driven analysis. J Med Virol.

[7] Berger D, Herkenhoff K, Mongey S (2020). An SEIR Infectious Disease Model with Testing and Conditional Quarantine. National Bureau of Economic Research.

[8] Linka K, Peirlinck M, Sahli Costabal F, Kuhl E (2020). Outbreak dynamics of COVID-19 in Europe and the effect of travel restrictions. Comput Methods Biomech Biomed Engin.

[9] Dur-e-Ahmad M, Imran M (2020). Transmission dynamics model of coronavirus COVID-19 for the outbreak in most affected countries of the world. Int J Interact Multimed Artif Intell.

[10] Hubbs C (2020). Social distancing to slow the coronavirus. https://towardsdatascience.com/social-distancing-to-slow-the-coronavirus-768292f04296.

[11] De Falco I, Della Cioppa A, Scafuri U, Tarantino E (2020). Coronavirus Covid-19 spreading in Italy: optimizing an epidemiological model with dynamic social distancing through differential evolution [Preprint]. arXiv.

[12] He X, Lau EHY, Wu P (2020). Temporal dynamics in viral shedding and transmissibility of COVID-19. Nat Med.

[13] Jones JH (2020). Notes on R0. Notes on R0 [Internet.

[14] Heffernan J, Smith R, Wahl L (2005). Perspectives on the basic reproductive ratio. J R Soc Interface.

[15] Backer JA, Klinkenberg D, Wallinga J (2020). Incubation period of 2019 novel coronavirus (2019-nCoV) infections among travellers from Wuhan, China, 20-28 January 2020. Eurosurveillance.

[16] Alsofayan YM, Althunayyan SM, Khan AA, Hakawi AM, Assiri AM (2020). Clinical characteristics of COVID-19 in Saudi Arabia: a national retrospective study. J Infect Public Health.

[17] Zhu Y, Chen YQ (2020). On a statistical transmission model in analysis of the early phase of COVID-19 outbreak. Stat Biosci.

[18] Prem K, Liu Y, Russell TW, Kucharski AJ, Eggo RM, Davies N (2020). The effect of control strategies to reduce social mixing on outcomes of the COVID-19 epidemic in Wuhan, China: a modelling study. Lancet Public Heal.

[19] Pandey G, Chaudhary P, Gupta R, Pal S (2020). SEIR and regression model based COVID-19 outbreak predictions in India [Preprint]. arXiv.

[20] (2020). Saudi Arabia’s ruthless fight against coronavirus. https://www.sa.undp.org/content/saudi_arabia/en/home/library/saudi-arabia-s-ruthless-fight-against-coronavirus.html.

[21] Hale T, Webster S, Petherick A, Phillips T, Kira B (2020). Oxford COVID-19 government response tracker. Oxford covid-19 government response tracker. Blavatnik Sch Gov.

[22] Van Rossum G, Drake FL (2011). The Python Language Reference Manual. https://dl.acm.org/doi/book/10.5555/2011965.

[23] Kluyver T, Ragan-Kelley B, Pérez F (2016). Jupyter Notebooks-a publishing format for reproducible computational workflows. Positioning and Power in Academic Publishing: Players, Agents and Agendas.

